# Cortical Functional Domains Show Distinctive Oscillatory Dynamic in Bimanual and Mirror Visual Feedback Tasks

**DOI:** 10.3389/fncom.2019.00030

**Published:** 2019-05-09

**Authors:** Salim M. H. Al-Wasity, Frank Pollick, Anna Sosnowska, Aleksandra Vuckovic

**Affiliations:** ^1^Rehabiliation Engineering Lab, Biomedical Engineering Research Division, University of Glasgow, Glasgow, United Kingdom; ^2^Department of Computer Science, University of Wasit, Kut, Iraq; ^3^School of Psychology, University of Glasgow, Glasgow, United Kingdom

**Keywords:** mirror-visual feedback, EEG, independent components analysis, measure projection analysis, event related synchronization/desynchronization, bimanual movement

## Abstract

It is believed that Mirror Visual Feedback (MVF) increases the interlimb transfer but the exact mechanism is still a matter of debate. The aim of this study was to compare between a bimanual task (BM) and a MVF task, within functionally rather than geometrically defined cortical domains. Measure Projection Analysis (MPA) approach was applied to compare the dynamic oscillatory activity (event-related synchronization/desynchronization ERS/ERD) between and within domains. EEG was recorded in 14 healthy participants performing a BM and an MVF task with the right hand. The MPA was applied on fitted equivalent current dipoles based on independent components to define domains containing functionally similar areas. The measure of intradomain similarity was a “signed mutual information,” a parameter based on the coherence. Domain analysis was performed for joint tasks (BM and MVF) and for each task separately. MVF created 9 functional domains while MB task had only 4 functionally distinctive domains, two over the left hemispheres and two bilateraly. For all domains identified for BM task alone, similar domains could be identified in MVF and joint tasks analysis. In addition MVF had domains related to motor planning on the right hemisphere and to self-recognition of action. For joint tasks analysis, seven domains were identified, with similar functions for the left and the right hand with exception of a domain covering BA32 (self-recognition of action) of the left hand only. In joint task domain analysis, the ERD/ERS showed a larger difference between domains than between tasks. All domains which involved the sensory cortex had a visible beta ERS at the onset of movement, and post movement beta ERS. The frequency of ERD varied between domains. Largest difference between tasks existed in domains responsible for the awareness of action. In conclusion, functionally distinctive domains have different ERD/ERS patterns, similar for both tasks. MVF activates contralateral hemisphere in similar manner to BM movements, while at the same time also activating the ipsilateral hemisphere. Significance: Following stroke cortical activation and interhemispheric inhibition from the contralesional side is reduced. MVF creates stronger ipsilateral activity than BM, which is highly relevant of neurorehabilitation of movements.

## Introduction

A specific type of mirror therapy, which is based on mirror-visual feedback (MVF), has been used for the rehabilitation of different forms of neuropathic pain (Matthys et al., 2009; Deconinck et al., 2014), and for the rehabilitation of movement in stroke patients (Michielsen et al., 2011). One of the therapeutic roles of MVF is to restore an interrupted efferent—afferent loop. In order to achieve the MVF illusion, a mirror is placed in a mid-sagittal plane between the intact and the affected limb, so that the reflection of the intact limb in a mirror is superimposed onto the affected one (Ramachandran and Rogers-Ramachandran, 1996). Any movement of the intact limb in front of the mirror causes the visual perception of two symmetrical, simultaneous movements of both limbs (Feltham et al., 2010; Deconinck et al., 2014). The overt movement of the limb in front of the mirror is accompanied by the overt or covert movement of the limb behind the mirror (in a mirror box).

It has been suggested that the mechanisms that underlie MVF are related to mismatched integration between vision, tactile sensation and proprioception (Hunter, 2003; Egsgaard et al., 2011). Another hypothesis has suggested that the mirror neuron system (Rizzolatti et al., 2001) may play a role in the mechanism of mirror therapy (Rosén and Lundborg, 2005; Yavuzer et al., 2008), although a systematic overview of MVF studies failed to confirm this hypothesis (Deconinck et al., 2014). Other theories have suggested that mirror therapy is a type of motor imagery, which creates visual feedback of the imagined limb movement (Stevens and Stoykov, 2003).

Movement visual illusion research studies which are based on different imaging modalities, such as functional Magnetic Resonance Imaging (fMRI), Magnetoencephalography (MEG) and Electroencephalography (EEG) (Deconinck et al., 2014) have reported increased activation of the motor cortex (M1) contralateral to the limb behind the mirror, compared with a unilateral limb movement (Touzalin-Chretien and Dufour, 2008; Tominaga et al., 2009; Touzalin-Chretien et al., 2009; Praamstra et al., 2011; Hadoush et al., 2013; Wang et al., 2013; Diers et al., 2015), along with the increased activation of areas involved with the allocation of attention and cognitive control (dorsolateral prefrontal cortex, posterior cingulate cortex, S1 and S2, precuneus) (Deconinck et al., 2014). Also, activation of the visual cortex ipsilateral to the moving hand has been noted during unimanual movement with a mirror (Dohle et al., 2004; Wang et al., 2013). Based on these findings, it was suggested that MVF influences areas related to perceptuo-motor processes; areas associated with the mirror system and processes related to the modulatory effect of the motor network (Deconinck et al., 2014).

While the mirror box illusion inherently involves movements of both limbs, most previously mentioned studies on able-bodied people compared the neural mechanism of MVF with a comparable mechanism during a unimanual task. In able-bodied people, cortical activation during MVF should resemble bimanual movements. To address this issue, Butorina et al. (2014) compared MVF (covert movement only) with bimanual symmetrical movement. They analyzed the topographical maps of surface cortical activity in several frequency bands (2–7, 10–25, and 55–85 Hz), and this demonstrated delta and gamma band synchronization and alpha/beta band desynchronization during both real and MVF overt movement. A detailed time-frequency analysis was however provided only for the primary motor cortex of the right and the left hand, neglecting areas responsible for a visuo-spatial integration.

The analysis of MVF phenomena has so far been focused on either fMRI or PET analysis of functional areas of cortex, or on the EEG analysis of dynamic phenomena in a time-frequency domain. In this study, we apply a recently developed Measure Projection Analysis (MPA) of EEG signals (Bigdely-Shamlo et al., 2013), which defines cortical areas involved in both tasks based on a probabilistic representation of equivalent dipoles based on independent components. Unlike k-mean clustering methods, it does not assume any shape of domains and even allows disconnected areas to be assigned to the same domain. In this study, we analyze functional domains for BM and MVF separately and we also perform domain analysis for joint tasks. We use event-related synchronization/desynchronization (ERS/ERD) (Pfurtscheller and Lopes da Silva, 1999) as a measure of intra-domain functional connectivity in domains common for both tasks. In this way, we compare the time-frequency dynamic of brain activity not only between real bimanual tasks and BMF tasks, but also between functionally distinctive domains over the whole cortex. We demonstrate characteristic, domain-specific patterns of ERS/ERD.

## Materials and Methods

### Participants

Fourteen right handed, able-bodied participants (age 32.85 ± 5.77, 13 male and 1 female) participated in the study. Participants' handedness was tested using the Edinburgh Handedness Inventory (Oldfield, 1971). Participants signed the informed consent form prior to taking part in the study. The study was approved by the University of Glasgow, College of Science and Engineering Ethical Committee. A member of research team provided a written consent to publish her photo.

### Experimental Design

**Figure 1:**
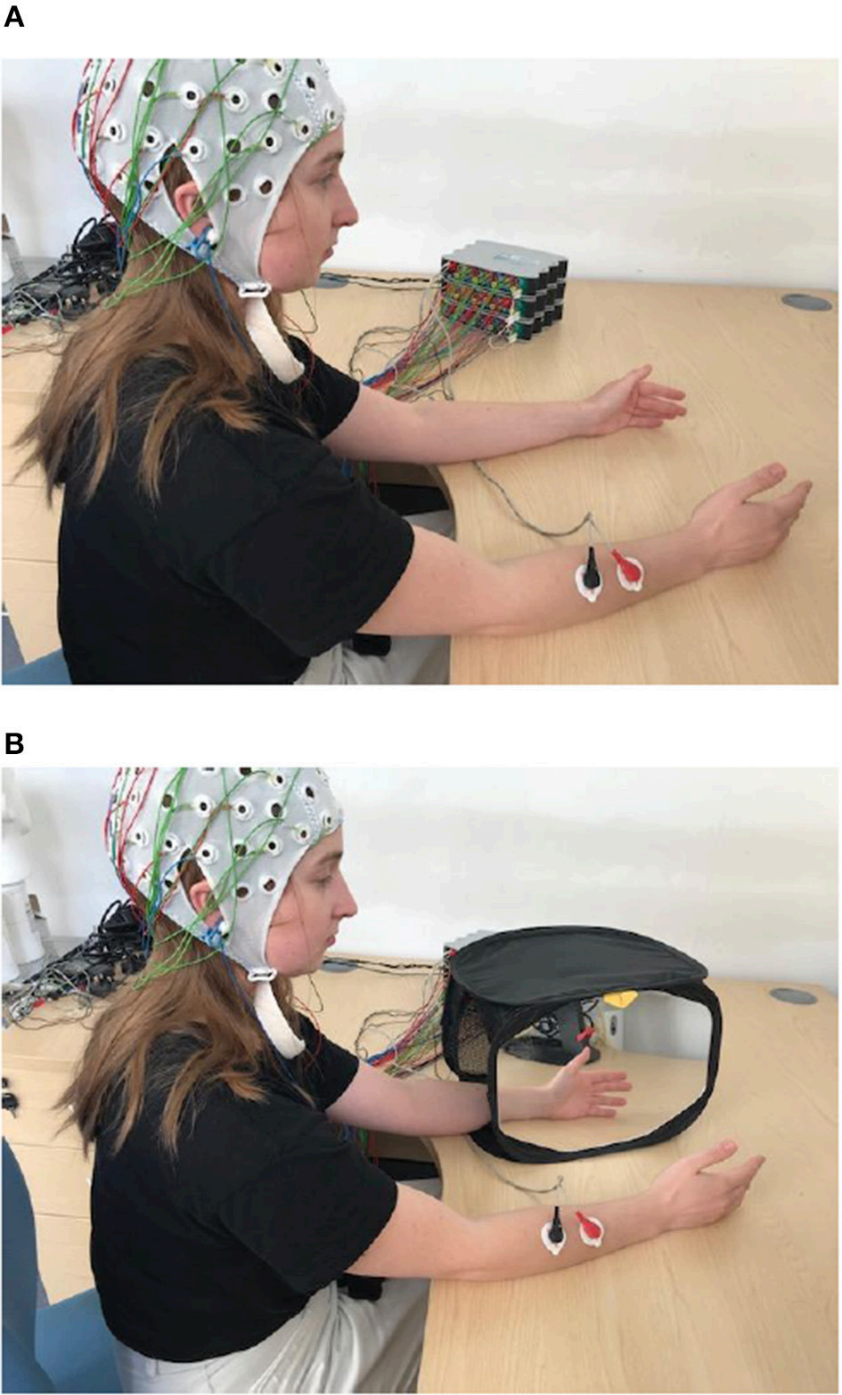
Experimental paradigm for BM **(A)** and for MVF task **(B)**. In BM participant is waving synchronously with both hands (synchronous hand flexion) and with right hand only for MVF task. Written informed consent has been obtained for the publication of this image.

Participants sat comfortably on a chair, with both hands resting on a desk. Their palms were placed to face each other, ~30 cm apart. Subjects were asked to perform two tasks; the first was a brisk bimanual hand and wrist flexion (BM task) and the second was the same movement performed with the dominant (right) hand only, using a MVF paradigm (Figure 1). In this paradigm, the non-dominant (left) hand was placed inside a mirror box, behind the mirror, and the dominant one was placed in front of the mirror, being visible to the participant. Participants were instructed to watch the reflection of their dominant hand in the mirror while performing movements, which created the illusion of movement of the non-dominant hand. All participants experienced illusion of movement. In 3 participants with strongest movement illusion, the movement of the dominant hand was occasionally accompanied with an overt movement of the non-dominant hand. We did not attempt to suppress these movements, as that might have influenced the intensity of the visual illusion.

Participants were instructed to wait ~10 s in between the two movements, so that sufficiently long baseline activity could be recorded for further data analysis. Trial sessions preceded the experiment, in order to familiarize the participants with self-paced movement, and to establish the pace of the task.

The experiment was divided into sub-sessions, with a break in between sub-sessions to avoid fatigue. Each sub-session consisted of 10–12 trials, resulting in 80–90 trials per task. Participants first performed the bimanual task, then the unimanual task.

### Data Recording

Participants' EEG and EMG were recorded using three cascading universal bio-signal amplifiers (USBAmp, Guger technologies, Austria), with separate ground and references for EEG and EMG measurements. Both EEG and EMG were sampled with the same frequency at 1,200 Hz and filtered with an IIR Butterworth notch filter (5th order) at 50 Hz; this was integrated into the amplifier. EEG signals were recorded using 44 surface electrodes according to international 10-10 electrode standard position (Jurcak et al., 2007). The impedance of the EEG electrodes was kept under 5 kΩ and the signal was filtered online with a band-pass IIR Butterworth filter (5th order), integrated into the amplifier, with a cut-off frequency of 2–100 Hz. The left earlobe was used as a reference and the right one as the ground. Two bipolar EMG channels were recorded using surface electrodes positioned over the right and left hand flexor muscles. The EMG signal was filtered online with a band-pass IIR Butterworth filter, integrated into the amplifier, with a cut-off frequency at 5 and 500 Hz.

### Data Analysis

The Measure projection analysis consisted of the following major steps (Bigdely-Shamlo et al., 2013). In both tasks, the onset of EEG movement-related activity was determined with respect to the right hand EMG signal. The EMG signal was rectified and smoothed using a moving average filter of 5th order. A threshold for the onset and offset of the EMG activity was set to mean+2STD (Moore et al., 2000). For each single subject, continuous EEG of both tasks was concatenated for further processing. An EEGLAB toolbox v.13.3.2b with plugins for performing clustering based on measure projection analysis (Delorme and Makeig, 2004) was used to process the EEG data. EEG epochs were extracted from continuous EEG recording with respect to the onset of movements defined by the EMG of the right hand for both tasks. For a movement onset detected at *t* = 0 s, an EEG epoch started at *t* = –4 s and ended at *t* = 3 s, providing a 7 s long recording. The epoched EEG was down-sampled to 300 Hz, filtered using Windowed-Sinc Band-pass FIR filter of 1560th order with a cut-off frequency of 1–45 Hz using a Blackman window (Smith, 2002), and re-referenced to the average reference.

**Figure 2:**
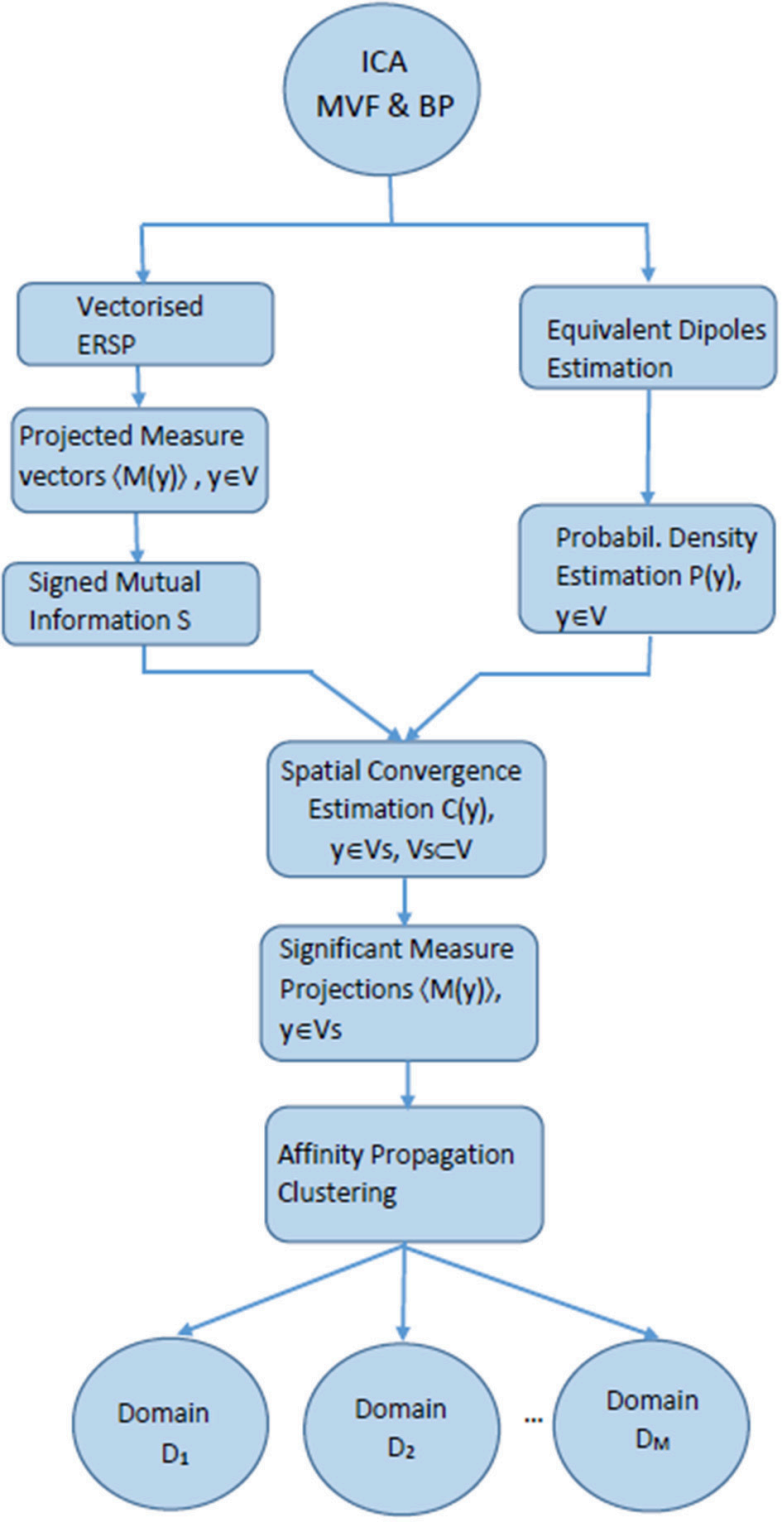
Flow chart explaining MPT algorithm with ERSP as projected measures.

The Measure projection analysis consisted of the following major steps (Bigdely-Shamlo et al., 2013) (Figure 2):

1. Independent component analysis decomposition of EEG signals for noise removal and for subsequent dipole localization followed by the calculation of equivalent dipoles.
2. Calculation of the chosen dynamic measure (Event related spectral perturbation, Makeig, 1993) and spatial smoothing of the measure for the equivalent dipole-located ICs.
3. True Measure projection analysis, i.e., defining the subspaces of brain voxel locations with the significant IC measures of similarity.
4. Affinity propagation clustering (Frey and Dueck, 2007), i.e., creating spatial brain voxel domains which exhibit sufficient differences in inter-domain correlation.

All calculations and group analyzes were performed in EEGLab using MPT toolbox. EEGLAB supports processing across sessions and subjects using a STUDY framework (Delorme et al., 2011). A STUDY structure was created using EEGLAB for multi-subject data analysis. The STUDY had one group (14 subjects) with two conditions, corresponding to the two motor tasks.

Following MPT analysis, an additional step was performed to compare between ERS/ERD of different conditions.

#### Independent Component Analysis and Source Localization

EEG artifacts (physiological and non-physiological) were removed by calculating independent components (IC) and removing components associated with noise before returning to the EEG domain. The Infomax Independent Component Analysis (ICA) method was used (Bell and Sejnowski, 1995), with the extended-ICA algorithm (Lee et al., 1999) which extracts the mixed sub-Gaussian and super-Gaussian sources effectively. Bad components were discarded based on a scalp map distribution and a power spectral density.

The locations of dipoles were determined for each remaining component based on the boundary element model MNI (Acar and Makeig, 2010). The localization of brain sources is known as an inverse problem (Edelvik et al., 2009; Daou and Labeau, 2014). Most of the IC scalp maps have nearly the same projection of a single equivalent dipole; therefore a single dipole location procedure was used to estimate the location of the ICs (Zou et al., 2006). This reduces the uncertainty of dipole localization as compared to dipoles derived from EEG, which present a mixture of many sources. A co-registration between channel locations and head model surface was performed to align the dataset channels' locations to a three-shell boundary element head template model montage. ICs of each subject were chosen if they were inside the brain volume and they had a residual variance (RV) <15%.

Finally, the ICs dataset was separated into two sets corresponding to the two tasks.

#### Event Related Spectral Perturbation

Event related spectral perturbation (ERSP) is a measurement tool that is used to analyze event-related EEG dynamics. This is an extended version of ERS/ERD which enables simultaneous analysis over a range of frequencies. It measures relative power changes of an EEG channel or an IC component in certain frequency bands during a dynamic task with respect to the baseline period before the task (Makeig, 1993). The ERSP was computed for a frequency range of 3–40 Hz using a Morlet wavelet with 3 cycles for the lowest frequency and 20 cycles for the highest frequency for all the selected ICs of all the subjects. The ERSP was calculated as power changes in decibels with reference to a baseline period (from t_1_ = −3.4 to t_2_ = −2.4 s). This period, quite far from the onset of EMG was selected to avoid a period of motor planning. Statistical significance of the ERS/ERD values was determined by applying a t-percentile bootstrap algorithm (Graimann et al., 2002) with a significance level α = 0.05.

ERS/ERD was presented in a logarithmic form in figures as it provides better visibility of ERS/ERD of alpha sensory-motor rhythms. To compare between means of two variables (measure projections based on ERS/ERD) a non-parametric permutation test, based on resampling, was implemented in EEGLAB with a significance level set to *p* = 0.05. To compare between ERS/ERD of two different conditions, a common baseline period was calculated. Correction for multiple comparisons was performed using the Holm-Bonferroni method (Holm, 1979).

While ERSP give an M × N matrix with M time and N frequency elements, for MPA analysis this matrix is vector, i.e., presented as an 1 × (M × N) vector. For visualization purposes only, this vector was transformed back to M × N matric.

Each IC is presented by its *measure vector* (vectorized ERSP) and a corresponding equivalent dipole.

#### Measure Projection Analysis

This is a crucial step which defines brain areas which contain ICs with significant similarities in projected measure vectors (i.e., projected vectorized ERSP).

Instead of representing equivalent dipoles of independent components as points, MPT represents each dipole with a 3D Gaussian with a density *P(y)* and with a standard deviation set to 12 mm (a user defined value, recommended in Bigdely-Shamlo et al. (2013), thus covering an area with a diameter of 36 mm.

Based on the density, it calculates the estimated (or “projected measure value”) *M*(y) of a certain experimental condition, at brain locations *y* spanning a regular 8 mm spacing grid:

(1) $$\begin{array}{l} E\left\{M\left(y\right)\right\}=\langle M\left(y\right)\rangle =\frac{\overset{n}{\underset{i=1}{\sum }}P_{i}\left(y\right)M_{i}}{\overset{n}{\underset{i=1}{\sum }}P_{i}\left(y\right)}=\overset{n}{\underset{i=1}{\sum }}\overset{-}{P}\left(y\right)M_{i} \end{array}$$

Where $\overset{-}{P}\left(y\right)$ (with a property $\overset{n}{\underset{i=1}{\sum }}{\overset{-}{P}}_{i}=1$) is a probability that the estimated location of measure vector *M*_*i*_*(y*) is truly *M(y)*. *M*_*i*_ is a vectorized ESRP corresponding to *IC*_*i*_ while 〈M (y)〉 is its “projection” on 3D Gaussian at voxel y. Once projected measures M (y) are obtained for each voxel, it is necessary to estimate their probability distribution, i.e., to identify brain areas, i.e., “neighborhoods” which exhibit statistically significant similarities in this measure.

Next, for each pair of dipoles *P*_*i*_*(y)* and *P*_*j*_*(y)*, it calculates the degree of similarity *S*_*i, j*_, which in this case presents “a signed mutual information” (Darbellay and Vajda, 1999), a measure based on correlation coefficient (*CORR*) between pair wise *IC* measure vectors (*ERSP*_i_ and *ERSP*_*j*_)

(2) $$\begin{array}{l} S=\frac{1}{2}sign\;\left(CORR\right)\log_{2}\left(\frac{1}{1-CORR^{2}}\right)\left(bit/sampe\right) \end{array}$$

Based on this, convergence *C(y)* was calculated.

(3) $$\begin{array}{l} C\left(y\right)=E\left\{S\left(y\right)\right\}=\frac{\overset{n}{\underset{i=1}{\sum }}\overset{n}{\underset{j=1,j\neq i}{\sum }}P_{i}\left(y\right)P_{j}\left(y\right)S_{i,j}}{\overset{n}{\underset{i=1}{\sum }}\overset{n}{\underset{j=1,j\neq i}{\sum }}P_{i}\left(y\right)P_{j}\left(y\right)} \end{array}$$

*C(y)* identifies brain areas, i.e., “neighborhoods” which show statistically significant similarities in estimated measure vectors 〈M (y)〉. C(y) has higher values for homogeneous (similar) areas. The significance of the similarity (*p*-values) is determined based on bootstrap statistics. This tests against the null hypothesis that 〈M (y)〉 (i.e., estimated ERSP) are produced by a random set of measure vectors with a statistical significance set to *p* = 0.05.

#### Affinity Propagation Clustering

Previous step only defines one global area of significant similarities. Affinity Propagation Clustering method (Frey and Dueck, 2007) was applied to cluster similar estimated projection measure vectors 〈M (y)〉 into separate spatial domains. Affinity Propagation Clustering determines the granularity of regions without changing the values of the parameters calculated in the previous step. In this study the maximum allowed correlation between domains was set to 0.9, meaning that data points between different domains were not allowed to have a higher correlation (i.e., be more similar) than 0.9.

This clustering method has the following properties:

1. It does not require prior knowledge about the number of clusters.
2. It determines outliers.
3. It creates clusters/domains which do not have a fixed geometric shape (e.g., sphere in k-mean clustering) and even allows spatially disconnected areas to be assigned to the same domain, which comprises highly functionally connected areas.
4. Because the MPA method is based on the probabilistic representation of dipole locations (rather than presenting equivalent dipoles of independent components as points), it is not necessary that IC of each single subject contribute to each domain (Bigdely-Shamlo et al., 2013).

#### Comparing Projected Estimated Measures Between Two Conditions

Projected estimated measures defined over significant brain areas C(y) are separated in two groups, MVF and BP. For a condition *c*, a weighted-mean measure *W(d,c)* across all ν voxels in domain *d* is:

(4) $$\begin{array}{l} W\left(d,c\right)=\frac{\sum_{i=1}^{\nu }M\left(c,i,d\right)\cdot D\left(i\right)}{\sum_{i=1}^{\nu }D\left(i\right)} \end{array}$$

(5) $$\begin{array}{l} D\left(i\right)=\overset{n}{\underset{j=1}{\sum }}P_{j}\left(i\right) \end{array}$$

Above, *n* is the number of component dipoles for a certain condition and *P*_*j*_*(i)* is the model probability that dipole *j* is actually at domain voxel *i*.

**Figure 3:**
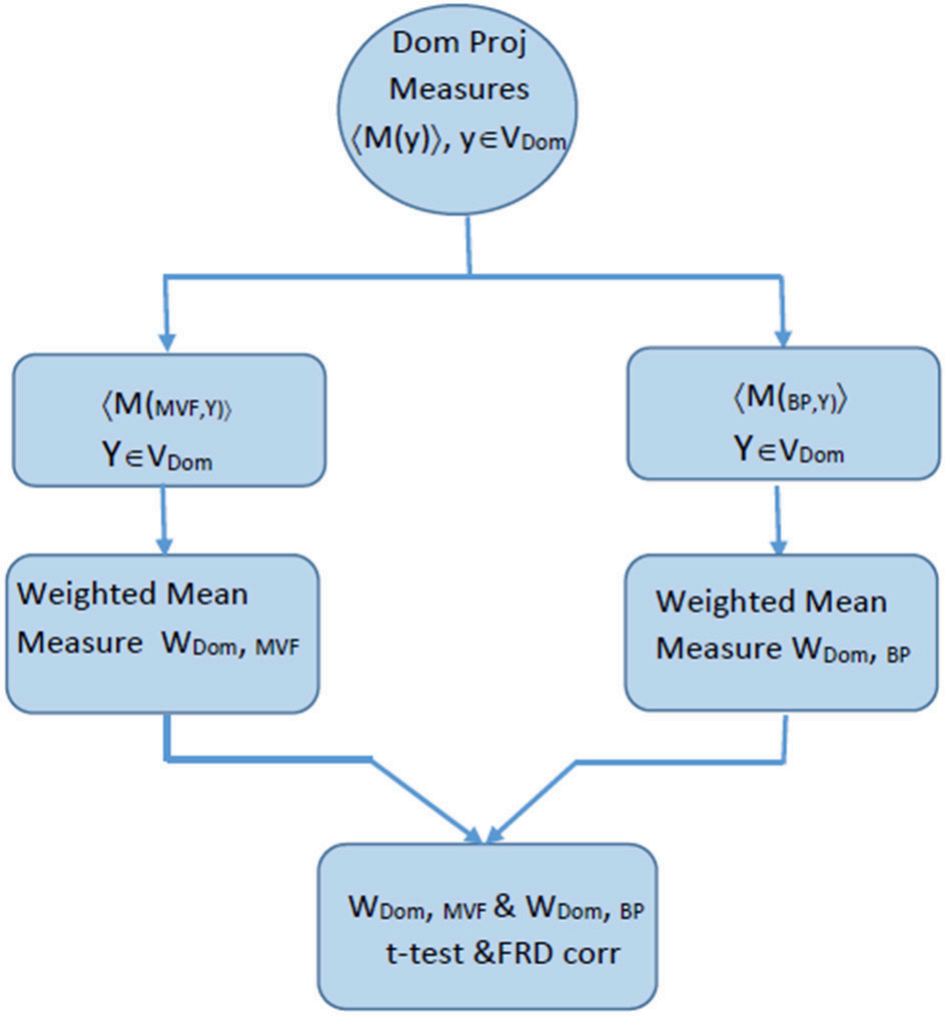
Flow chart explaining the process of comparing ERSP for BM and MVF within one domain, that was calculated based on the algorithm described in Figure 2.

Once W(d,c) values were obtained for both condition, a two-tailed student *t*-test was applied to reveal a statistically significant differences between conditions within a domain. All regions with p > 0.05 are shaded in ERSP graphs in the section Results. The flow diagram of the procedures in shown in Figure 3.

## Results

We first analyzed the spatial location and functional relevance of each domain. Following this, we created ERSP maps for both tasks in each domain, and compared them between tasks, within the same domain, and between functionally different domains.

### Joint Tasks Analysis of Domains

**Figure 4:**
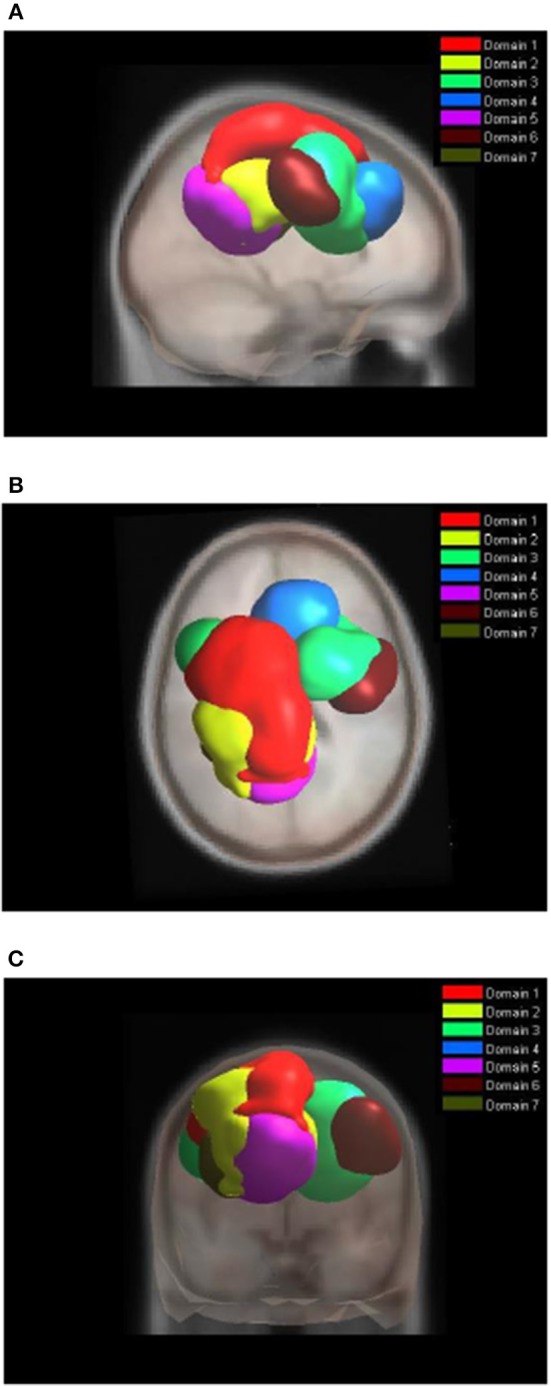
Location of domains relative to each other. **(A)** Sagittal view; **(B)** Top view; **(C)** Posterior view.

**Figure 5:**
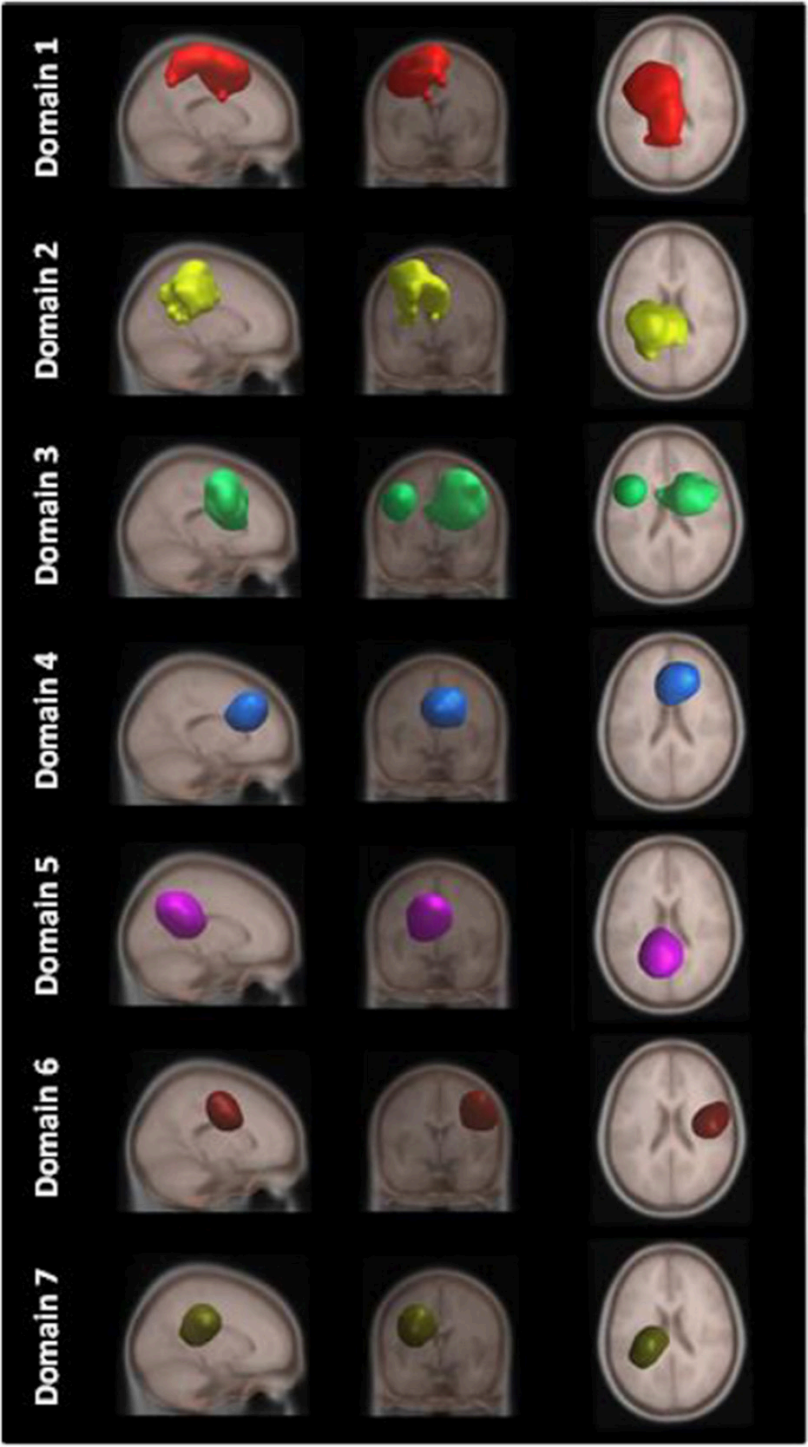
MPT domains obtained by clustering statistically significant ERS/ERD measures of similarity.

Figure 4 shows areas of the brain which showed a highly significant similarity, by means of the analysis described in section Measure Projection Analysis. The area is divided into 7 domains and Figure 1 shows their location relative to each other. Figure 5 presents sagittal, top and posterior view of each separate consistent domains. Note that the domains were created based on ERS/ERD values from both motor tasks, and contained dipoles of both tasks. Table 1 lists the anatomical location and Brodman Areas (BA) of each domain (Brodmann, 2006). The numbers in brackets show the probability of the mentioned domain occurring in that BA/anatomical location. It should be noted that although one BA might belong to several domains, those domains do not overlap. This means that if one BA is attributed to two domains, different portions of that BA belong to different domains. Joint domain analysis allowed for comparison of ERS/ERD maps between motor tasks.

**Table 1:** The anatomical location and BA of the domains for BM and MVF tasks together.

**Domain**	**Anatomical areas**	**BAs**	**Subjects**
1	L superior frontal gyrus (0.37) L precentral gyrus (0.20) L postcentral gyrus (0.10) L superior parietal gyrus (0.09) L middle frontal gyrus (0.07) L precuneus (0.05)	BA 6 (0.44) premotor and supplementary motor BA 7 (0.21) somatosensory association BA 4 (0.15) primary motor BA 5 (0.10) somatosensory association BA 3 (0.06) primary somatosensory	13
2	L postcentral gyrus (0.34) L superior parietal gyrus (0.33) L precentral gyrus (0.13) L precuneus (0.05)	BA 40 (0.19) spatial and semantic processing BA 7 (0.17) somatosensory association BA 5 (0.15) somatosensory association BA 3 (0.14) primary somatosensory BA 31 (0.12) BA 4 (0.11) primary motor BA 2 (0.05) primary somatosensory	13
3	R middle frontal gyrus (0.38) R superior frontal gyrus (0.30) R precentral gyrus (0.13) l middle frontal gyrus (0.12)	BA 6 (0.67) premotor and supplementary motor BA 24 (0.11) BA 32 (0.07) BA 9 (0.06)	8
4	R superior frontal gyrus (0.51) L superior frontal gyrus (0.19) R middle frontal gyrus (0.17) R cingulate gyrus (0.08)	BA 32 (0.36) BA 8 (0.33) includes frontal eye fields and lateral and medial supplementary motor area (SMA) BA 6 (0.17) premotor and supplementary motor BA 24 (0.07) BA 9 (0.06)	10
5	L superior parietal gyrus (0.38) L precuneus (0.30) L cingulate gyrus (0.17) R precuneus (0.07)	BA 31 (0.56) BA 7 (0.25) somatosensory association BA 23 (0.07) BA 30 (0.06)	9
6	R precentral gyrus (0.65) R postcentral gyrus (0.31)	BA 4 (0.32) primary motor BA 3 (0.30) primary somatosensory BA 6 (0.26) premotor and supplementary motor BA 2 (0.05) primary somatosensory	5
7	L postcentral gyrus (0.34) L superior parietal gyrus (0.21) L supramarginal gyrus (0.20) L angular gyrus (0.08) L cingulate gyrus (0.08)	BA 31 (0.39) BA 40 (0.27) spatial and semantic processing BA 13 (0.14) inferior insula BA 2 (0.11) primary somatosensory BA 3 (0.06) primary somatosensory	4

*Numbers between brackets show the probability of the mentioned domain occurring in that area*.

The first and largest domain, D1, was located in the left hemisphere, contralateral to the right, active hand. It contained areas that were responsible for motor planning (premotor cortex BA6) and execution (primary motor cortex BA4), as well as somatosensory association areas (BA5,7) in the posterior parietal cortex and, to a smaller degree, the primary somatosensory cortex (BA3).

The second domain, D2, was also located in the left hemisphere and comprised functionally related areas covering the superior (BA5,7) and posterior (BA40) parietal cortices, with additional contributions from the primary somatosensory cortex S1 (BA2,3) and primary motor cortex M1 (BA4). It shared some similar BAs with domain 1, with the main difference being a contribution from the secondary somatosensory cortex (BA40), indicating the functional relevance of this domain for visuo-spatial integration (Bear et al., 2007).

The third domain, D3, contained two spatially disjointed domains. It was located predominantly on the right side, ipsilateral to the moving hand, but it also occupied a smaller portion of the left middle frontal gyrus (BA9). The largest part of this domain (67%) was located in BA6, the pre-and supplementary motor area of the left hand, responsible for movement planning (Bear et al., 2007). It also included parts of the central anterior cingulate cortex BA24 and BA32 on the right side.

The fourth domain, D4, was located predominantly on the right side of the frontal gyrus, but it bilaterally covered the superior frontal gyrus, and the right side of the middle frontal gyrus and cingulate gyrus. The largest part of this domain belonged to the right anterior cingulate cortex ACC (BA32), which is responsible for awareness of actions, and self-recognition (Devue et al., 2007) and to the pre- and supplementary motor cortices (BA6). One third of the domain is occupied by BA8, frontal eye fields (Vernet et al., 2014). It also included smaller portions of BA24 and BA9.

The fifth domain, D5, was predominantly located to the left, with a smaller part occupying the right side and including the precuneus. The two major BAs in this domain were BA31, located at the posterior cingulate cortex, and BA7 in the somatosensory association area.

The sixth domain, D6, was located on the right side and dominantly covered primary, pre- and supplementary motor cortex and primary somatosensory cortex, BA4, BA6, and BA3, respectively.

The seventh domain, D7, located in the left hemisphere largely covered the parts of BA31 not included in domain 5, as well as portions of BA40. It also included, to a smaller extent, the primary somatosensory cortex (BA2,3) on the left side.

### The Analysis of Domains for Individual Tasks

While the analysis of both tasks together enables comparison of ERS/ERD maps, the analysis of domains for each task separately reveals differences in spatial organization of functional domains. Nine functional domains for MVF task indicated larger complexity of MVF as compared to BM task which resulted in 4 functionally distinctive spatial domains.

**Figure 6:**
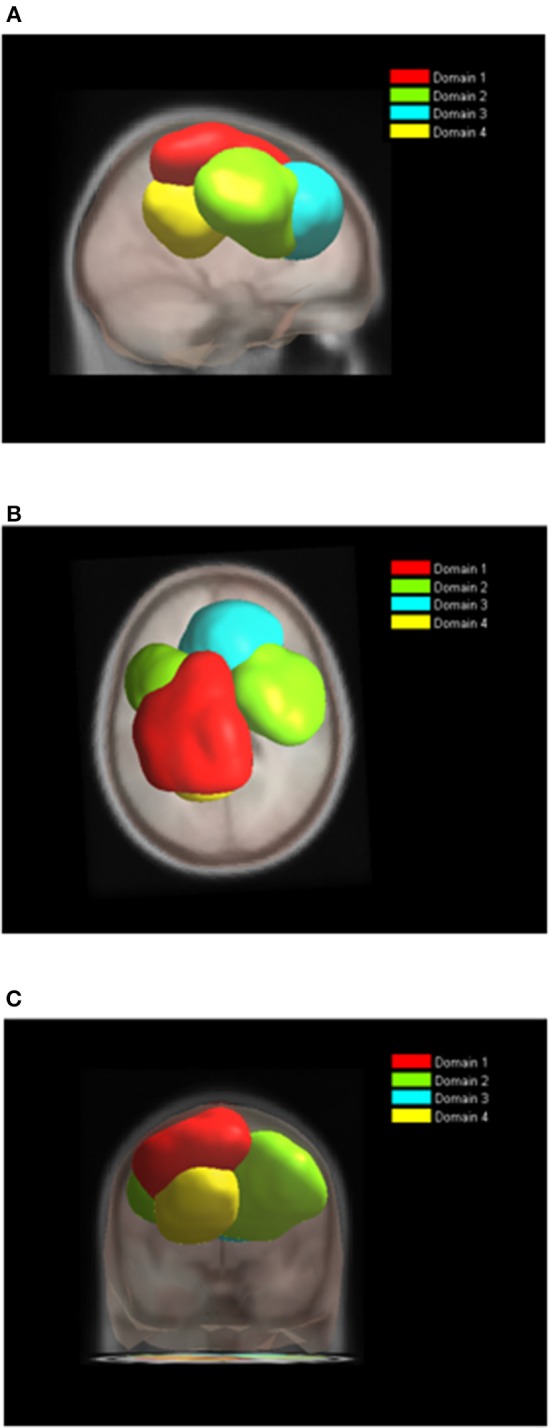
Location of domains relative to each other for BT tasks. **(A)** Sagittal view; **(B)** Top view; **(C)** Posterior view.

During BM (Table 2, Figure 6), the largest domain (D1_BM_) located on the left hemisphere, covered area responsible for motor planning and execution as well as somatosensory association areas. In largely corresponded to Domain 1 in analysis of both tasks together (section Joint Tasks Analysis of Domains). Domain D2_BM_ is located dominantly on the right hemisphere, with largest portion containing BA 6 responsible for motor planning as well as primary sensory and motor cortex. It is most similar to D3. Domain D3_BM_ covers mostly superior frontal gyrus bilaterally, including BA8 and BA32, similar to D4. Finally domain D4_BM_ is located on the left hemisphere over BA31 and BA7 and has similar spatial location as D5 in section Joint Tasks Analysis of Domains.

**Table 2:** The anatomical location and BA of the domains for BM task.

**Domain**	**Anatomical areas**	**BAs**	**Subjects**
1	L superior parietal gyrus (0.26) L superior frontal gyrus (0.20) L postcentral gyrus (0.18) L precentral gyrus (0.16)	BA 7 (0.26) BA 6 (0.21) BA 4 (0.13) BA 5 (0.13) BA 40 (0.13) BA 3 (0.08)	13
2	R middle frontal gyrus (0.29) R precentral gyrus (0.20) R superior frontal gyrus (0.15) L middle frontal gyrus (0.13) R postcentral gyrus (0.12)	BA 6 (0.49) BA 3 (0.12) BA 4 (0.11) BA 24 (0.06) BA 8 (0.05)	
3	R superior frontal gyrus (0.49) L superior frontal gyrus (0.22) R middle frontal gyrus (0.20)	BA 8 (0.36) BA 32 (0.26) BA 6 (0.10) BA 9 (0.15) BA 24 (0.06)	13
4	L superior parietal gyrus (0.37) L cingulate gyrus (0.24) L precuneus (0.22) L postcentral gyrus (0.07)	BA 31 (0.71) BA 7 (0.15) BA 23 (0.08)	8

*Numbers between brackets show the probability of the mentioned domain occurring in that area*.

**Figure 7:**
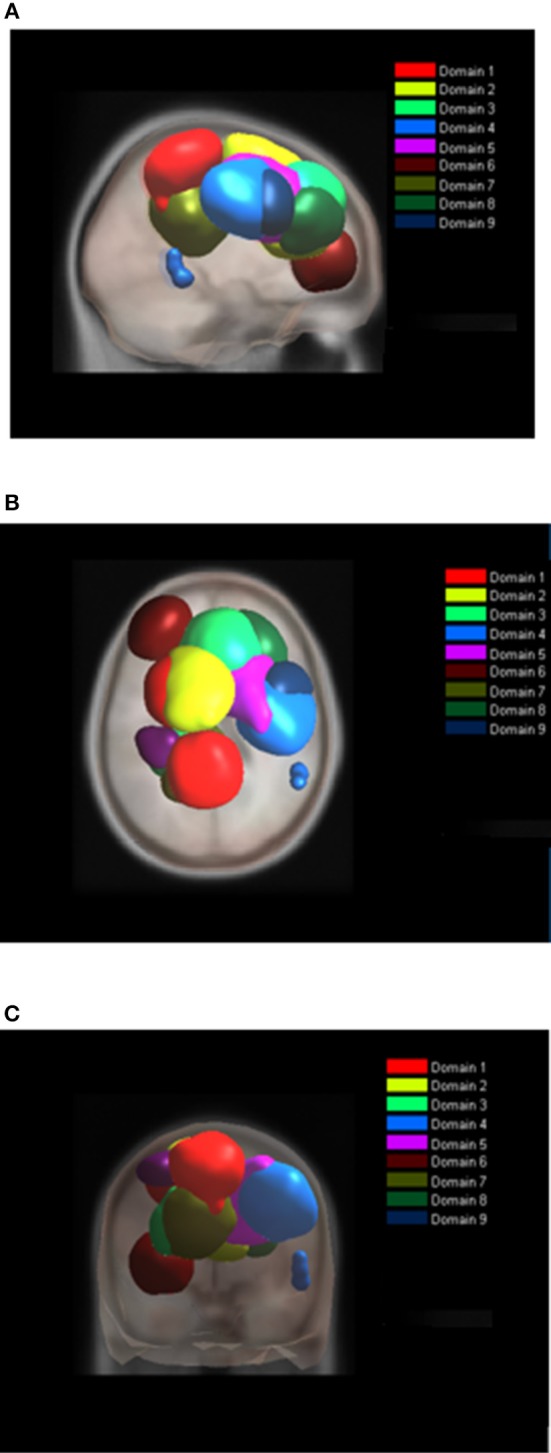
Location of domains relative to each other for MVF tasks. **(A)** Sagittal view; **(B)** Top view; **(C)** Posterior view.

For MVF nine functional domains were identified (Table 3, Figure 7). Domain D1_MVF_ is located on the left hemisphere and largely covered the same areas as D1 and D1_BM_. Domain D2_MVF_ covered bilaterally superior frontal gyrus (BA6) that is responsible for motor planning. Domain D3_MVF_ was located over the superior frontal gyrus and had a similar spatial location as D4 and D4_BM_. Domains D4_MVF_ and D5_MVF_ were both located over the right hemisphere. D4_MVF_ covered the sensory-motor cortex and was most similar to D6. Domain D5_MVF_ covered the premotor cortex BA6 as well as BA32 and BA24. It was most similar to D3, covering areas responsible for awareness of actions and self-recognition. Domains D2_MVF_ and D5_MVF_ covered similar area as D2_BM_. Domain D7_MVF_ was located over left prefrontal cortex (BA10 and BA46) responsible for sustained attention and working memory. Domain D7_MVF_ was also located over the left hemisphere and was most similar to D5. Domains D8_MVF_ and D9_MVF_ were both located over the right hemisphere. Largest portion of D8_MVF_ covered right BA9 which is amongst the others responsible for processing coordinate spatial relations (Slotnick and Moo, 2006), overriding automatic responses (Kübler et al., 2006), error detection (Chevrier et al., 2007) and inferring deduction from spatial imagery (Knauff et al., 2002). Most notable area present in several MVF domains was the BA6, including a domain which included only bilateral BA6, and domains which included BA6 on left or right hemispheres separately.

**Table 3:** The anatomical location and BA of the domains for MVF task.

**Domain**	**Anatomical areas**	**BAs**	**Subjects**
1	L superior parietal gyrus (0.34) L postcentral gyrus (0.17) L precuneus (0.16) R precuneus (0.09) L precentral gyrus (0.07) R superior parietal gyrus (0.07) R postcentral gyrus (0.07)	BA 7 (0.59) BA 6 (0.23) BA 4 (0.10)	13
2	L superior frontal gyrus (0.86) R superior frontal gyrus (0.11)	BA 6 (0.89)	10
3	R superior frontal gyrus (0.61) L superior frontal gyrus (0.26) R middle frontal gyrus (0.09)	BA 8 (0.43) BA 6 (0.26) BA32 (0.24)	13
4	R precentral gyrus (0.38) R postcentral gyrus (0.32) R middle frontal gyrus (0.16) R supramarginal gyrus (0.12)	BA 6 (0.27) BA 3 (0.24) BA 4 (0.21) BA 40 (0.12) BA 2 (0.10)	8
5	R superior frontal gyrus (0.45) R middle frontal gyrus (0.35) R precentral gyrus (0.10)	BA 6 (0.47) BA 32 (0.20) BA 24 (0.20) BA 8 (0.08)	8
6	L inferior frontal gyrus (0.45) L middle frontal gyrus (0.44) L lateral orbitofrontal gyrus (0.06)	BA 10 (0.67) BA 46 (0.23)	8
7	L superior parietal gyrus (0.33) L cingulate gyrus (0.30) L precuneus (0.22) L postcentral gyrus (0.06)	BA 31 (0.68) BA 7 (0.15) BA 23 (0.09)	8
8	R middle frontal gyrus (0.88) R superior frontal gyrus (0.09)	BA 9 (0.49) BA 8 (0.31) BA 32 (0.17)	
9	R middle frontal gyrus (0.72) R precentral gyrus (0.24)	BA 6 (0.70) BA 9 (0.19) BA 8 (0.09)	8

*Numbers between brackets show the probability of the mentioned domain occurring in that area*.

When analyzed individually, both BM and MVF tasks had similar spatial distribution of their largest domains D1_BM_ and D1_MVF._ In addition, their spatial location corresponded to the domain D1 in analysis of both tasks. Domains functionally related to motor planning could also be identified in all three cases (D3, D2_BM_, and D5_MVF_) as well as a domains related to the awareness of action (D4, D3_BM_, and D3_MVF_) and domains having the function of sensory sustained attention (D5, D_BM4_, and D_VMF7_). Table 4 shows brief functional description of each domain. It enables a brief overview of main similarities and differences between different domains in all three cases.

**Table 4:** Similar Domains in joint BT & MVF analysis and in the analysis of separate tasks.

**Function**	**Domain BM&MVF**	**D_**BM**_**	**D_**MVF**_**
Motor planning and execution, sensory-motor integration; left	1	1	1
Visuo-spatial integration; left	2	–	–
Motor planning; dominantly right	3	2	2&5
Awareness of action; bilateral	4	3	3
Somatosensory, sustained attention, working memory; left	5	4	7
Sensory-motor; right	6	–	4
Somatosensory and spatial integration; left	7	–	–
Sustained attention, working memory; left	–	–	7
Spatial relations; right	–	–	8
Motor planning; right	–	–	9

### The Analysis of Event Related Synchronization/Desynchronization

**Figure 8:**
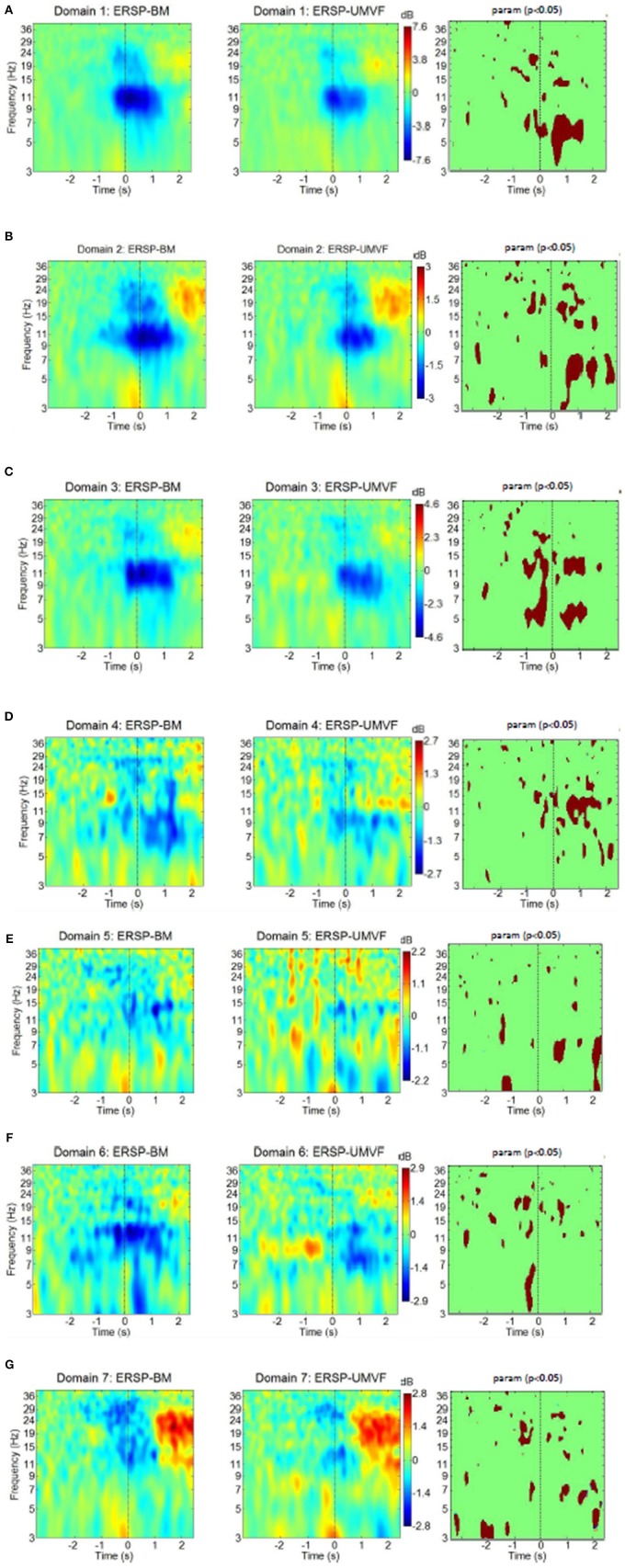
ERS/ERD of MPT domains for both tasks with their statistical test (column to the right) using parametric statistic with a *p*-value of 0.05, no correction for multiple comparison. BM-bimanual hand movement, MVF-unimanual hand movement with mirror. **(A–G)** present domains 1–7, respectively.

Figure 8 shows ERD/ERS maps of all seven domains for both tasks. Moment *t* = 0 s corresponds to the physical onset of movements as detected by EMG of the right hand. Statistically significant differences between the tasks were calculated based on permutation analysis (p = 0.05), and are shown in the column to the right. After applying a correction for multiple comparisons using the False Discovery Rate (FDR) method (Benjamini and Yekutieli, 2001), no statistically significant difference was found between the tasks in any domain. This is however quite a conservative test which may induce Type II error, as it does not account for spatial distribution of significant areas in time-frequency domain. From that reason we show statistically significant difference without FDR correction.

Domain 1, which was responsible for movement planning and execution, had strong ERD in the alpha band, and to some extent in the beta band. These ERDs started several hundred milliseconds before the onset of movement. This was followed by weak ERS in the beta band. ERD during movement execution was stronger in BM task in the theta band. Domain 1 had the strongest ERD of all domains, and was similar in both BM and MVF because it covers the motor cortex of the right hand. Its maximum ERD and ERS were twice as high as in the domain with the second strongest ERD/ERS (shown in the bars on the side).

Domain D2 had strong ERD in the alpha and the beta band, followed by beta ERS. The EDR was stronger n BM task during movement preparation in the beta band and during execution in the beta and theta bands. Domain 2 also had a strong ERS in the theta band, which relates to the physical onset of movement, thus probably having a proprioceptive contribution. Theta ERS has also been reported at the onset of visual or proprioceptive stimuli in experimental paradigms involving cue-based motor tasks (Reynolds et al., 2005). In the BM task, alpha ERD started earlier in domain 2 than in domain 1. In addition, judging by the maximal intensity of ERD and ERS (bars to the right of ERS/ERD maps), domain 2 had stronger beta ERS relative to alpha ERD than domain 1. This might be attributed to the contribution of somatosensory areas in domain 2, as it is believed that this phenomenon is related to the processing of somatosensory afferent input (Cassim et al., 2001).

Domain D3 had ERS/ERS similar to domain 1 in both tasks, though domain 3 was located in the right hemisphere. A large part of this domain comprises BA6, i.e., it is related to motor planning. The largest difference between BM and MVF tasks could be seen just before the physical onset of movement (at *t* = 0) across theta, alpha, and lower beta band, which is not surprising, because in MVF there was no planning of left hand movement. During movement execution difference between BM and MVF task persists in the lower beta and in the theta band, but not in the alpha band.

Domain D4 is largely located in BA32, and is responsible for the awareness of an action on the right side, so it is to be expected that ERS/ERD would demonstrate large differences between BM and MVF tasks in this domain. For BM, there was a widespread ERD over the alpha and beta band, while for the MVF, alpha ERD was accompanied by beta ERS. BM has stronger EDR in the alpha and lower beta bands. This unusual pattern for MVF task might reflect the influence of mismatched intentions of an action and the sensory feedback.

Domain D5 located over the left somatosensory hemisphere is characterized by weak ERD in the lower beta band in both tasks. It has stronger ERD for MVF during movement preparation in the theta band around *t* = −1 s and stronger ERS in the alpha and theta band following movement execution around *t* = 2 s.

Domain D6, located on the right side over M1, S1, and SMA is responsible for movement planning and execution of the left hand. Therefore, ERD could be detected during movement preparation only in the BM task, having significantly stronger ERD in the theta band. In the case of MVF, alpha ERD could be detected from 0.5 s after the onset of movement of the right hand.

Finally, domain D7, covering S1 and SII on the left side, was characterized by beta ERD followed by post movement beta ERS and theta ERS at the onset of movement. It has stronger ERD in the beta band during movement preparation for BM task. Following movement execution around *t* = 2 s MVF has stronger ERD in the theta band. The ERS/ERD response of this domain was similar to the response of domain 2. The main difference was a presence of beta ERD in domain 7, as opposed to alpha ERD in domain 2. Responses were similar for both tasks, probably because this domain characterizes the left hemisphere.

## Discussion

This study defined cortical domains using high intra-domain functional connectivity in three different scenarios (BM, MVF and joint BM and MVF tasks) to reveal tasks specific connectivity and connectivity common for both tasks. Analysis of separate tasks showed that while MVF covers all domains as BM tasks, it also includes additional domains related to motor planning and self-recognition of action. For joint tasks analysis, time-frequency responses of each tasks and for each single domain were presented. The analysis revealed differences in ERS/ERD between the tasks and between domains. Interestingly, for most domains the differences were more distinctive between domains than between tasks.

Previous MVF studies have often compared the movement of one hand (unimanual), in contrast with BM which includes both hands (Touzalin-Chretien and Dufour, 2008; Tominaga et al., 2009; Touzalin-Chretien et al., 2009; Praamstra et al., 2011; Hadoush et al., 2013; Wang et al., 2013; Diers et al., 2015). They have found significantly stronger cortical activity during MVF as compared to unimanual voluntary movements. From neuroimaging studies it is known that BM do not necessarily produce stronger activity than unimanual movement of the dominant hand. However BM are characterized by stronger intrahemispheric and interhemispheric correctively as measured by multi psychophysiologic interaction (mPPI) protocol (Szameitat et al., 2012). Connectivity between BA6 and BA40 is in particular prominent in BM tasks.

Previous neuroimaging studies have suggested three major functional networks involved in MVF: perceptuo-motor control, imitation of biological skills and modulatory effects of motor network (Deconinck et al., 2014). All of these networks were found in the MPA domain analysis. The novelty of the study is that it also present time-frequency analysis within these domains and compares it with similar analysis of BM task.

We will start the analysis by comparing domains D_BM_ and D_MVF_ with each other and with domains identified in a joint task analysis. In this study 4 domains for BM tasks alone were identified, 2 exclusively on the dominant left hemisphere indicating intrahemispheric connectivity and two over both hemispheres, indicating intra hemispheric connectivity. Domain D1_BM_ included areas related to motor planning (BA4,6) and perceptuo-motor control (BA5,7). It could be functionally attributed to the localization of objects in space, and to motor planning and execution (Bear et al., 2007). This domain comprised of both BA6 and BA40 confirming strong fronto-parietal connectivity in BM tasks, as suggested by Szameitat et al. (2012). Importantly, similar domain were identified for MVF and for analysis of the joint tasks (D1_MVF_ and D1). This shows that prominent fronto-parietal connectivity also exists in MVF and is of a comparable intensity, as measured by ERS/ERD.

Almost half of second largest domain D2_BM_ included BA6 (left and right frontal gyri) as well as smaller parts of M1 and S1 on the right hemisphere, and ACC (BA24), and BA8. Its functional role was motor planning. Similarly to D2_BM_, domain D3 in joint tasks covered dominantly the frontal gyrus bilaterally as well as the precentral gyrus on the right hemisphere. In MVF this areas were split in two domains, D5_MVF_ which also consisted almost 50% of BA6 over the right hemisphere and D2_MVF_ which consisted almost exclusively of BA6 bilaterally.

Domain D3_BM_ corresponded to D4 and to D3_MVF_. It contained BA32 responsible for the averseness of an action. This domain might not be seen in amputees or stroke patients who cannot move a paretic hand because they do not experience conflicting sensory (visual and proprioceptive) and motor information. This area is active is people who cannot suppress involuntary actions (Lhermitte, 1983). The rest of D3_BM_ corresponded to the area of frontal eye fields BA8 (Vernet et al., 2014) that reflects the visual input to motor tasks.

Domain D4_BM_ (BA7, BA31, and BA23) was related to the perceptual awareness of movement (Buckner et al., 2008) and to visuo-motor integration. A domain covering the same three BA in MVF task was D7_MVF_ and D5 in joint tasks domains.

Three domains were identified for MVF task, that were not present for BM task. Domain D4_MVF_, located in the right cortex was functionally related to motor learning as it involved the right cortex M1, pre-motor cortex and primary sensory cortex (BA4, BA6) (Bear et al., 2007; Merians et al., 2009) and sensory-motor integration (BA3). Activation of M1 ipsilateral to the active (right) hand is believed to play a crucial part in motor recovery in stroke patients practicing MVF (Deconinck et al., 2014). Similar domain D6 was also found in the joint domain analysis.

Another domain related to motor planning over the right hemisphere was D5_MVF_, consisting largely of BA6 with contribution from BA24, BA32, and BA8 and is similar to D3. Largest portion of domain D8_MVF_ covered right BA9 which is amongst the others responsible for processing coordinate spatial relations (Slotnick and Moo, 2006), overriding automatic responses (Kübler et al., 2006), error detection (Chevrier et al., 2007) and inferring deduction from spatial imagery (Knauff et al., 2002). This domain cannot be found in joint analysis. Finally Domain D7_MVF_ was located over the left prefrontal cortex (BA10 and BA46) responsible for sustained attention and working memory.

In summary, for all domains present for BM task similar domains were identified for MVF task. In MVF motor planning is in addition divided into functional unit over the left hemisphere, right hemisphere and bilaterally. During BM task, areas responsible for bilateral and right hemisphere motor planning are located within one domain. Additional functional domains which exist in MVF task only, related to sustained attention and to processing spatial relations, reflecting the nature of the MVF task which require visual engagement and result in sensation of movement of the left hand.

Joint task domain analysis showed that domains located in the right hemisphere covered the same functional areas as domains in the left hemisphere and in addition cover areas responsible for self-recognition of action. Two domains found in joint analysis were not directly comparable to any domain in the individual task analysis. These are domain D2 located over the left hemispheres including somatosensory association areas BA5 and BA7 as well as primary sensory and motor cortex (BA2, BA3, and BA4). All these BA are present within DBT and DMVF but not as one functional unit. Likewise, D7 located in the left hemisphere (BA31, BA40) became functionally independent unit only when both tasks are analyzed together. Right side BA6, premotor and supplementary motor area of left hand was the part of several functionally module, in domains D3 D4, and D6. It dominantly contributed to domain 3, the primary function of which was motor planning, but it also contributed to domain 4 with main function in self-recognition of action. Finally, together with BA3 and BA4, BA6 created a domain 6, responsible for motor planning, execution and sensory-motor integration (Bear et al., 2007). This confirms hypothesis of the indirect influence of self-recognition of an action (BA32), to motor execution (BA4) through motor planning (BA6) (Frith et al., 2000).

ERS/ERD were compared between domains in a joint task analysis only, as it was not possible to compare them directly between domains of separate tasks. However, based on the spatial similarity of functional domains in BM and MVF tasks, one can assume that ERS/ERD analysis of joint task truly reflects ERS/ERD differences between tasks. Most notable results was that each domain had a specific ERS/ERD pattern. Importantly, all domains which involved the sensory cortex had a visible post movement beta ERS and theta ERS at the onset of movement for both tasks. All three first domains (D1–D3) had strong alpha ERD. Domains D1 and D3, containing pre and supplementary motor cortices on the left and the right hemisphere (i.e., being functionally similar) had similar ERD/ER. Domains D4 and D5, which did not include the motor cortex, did not have as strong alpha ERD as the first three domains. In domain D5, located on in the left hemisphere ERD was very weak for both tasks, and present only after initiation of movement. Finally, the small domain D7 which covered S1 and SII on the left hemisphere, had beta ERD followed by strong beta ERS, and thus demonstrated ERD on a different frequency to domains which involved the motor cortex.

We also compared ERS/ERD between BM and MVF tasks. During preparation of movement, ERD was stronger in most domains, in particular in Domain 3, for BM taks. During execution of movement, ERD was stronger for BM task in Domains 1–4, while it was stronger for MVF task in Domains 5 and 7. Domains 1–4 covered motorplanning over both hemispheres. Domains 5 and 7 covered somatosenory cotex, sustained attention and spatial integration over the left hemisphere.

In this study we analyzed MVF with right, dominant hand, being the active hand in front of the mirror. A number of studies compared MVF of the dominant and non-dominant hand. Garry et al. (2005) found no difference in ipsilateral facilitation during MVF of the left and the right hand. However, a study observing real movements in able-bodied people found different strengths of reciprocal inter-hemispheric inhibitory effect exerted by the dominant and non-dominant hemispheres (Duque et al., 2007). They found that for the right active hand, the intermanual inhibition turns to intermanual facilitation closer to the movement onset, while for the non-dominant left hand inhibition remains. This indicates stronger intensity of activation of the ipsilateral cortex when right hand was active. Fritzsch et al. (2013) showed that MVF with either left or right active hand results in similar lateralization of activity in primary motor cortex (M1), but they found stronger activity in primary sensory cortex for left hand movements. Another study however found that the level of involuntary muscle activity (implying the involvement of M1) of the hand behind mirror also differ between dominant and non-dominant hand, being larger for the non-dominant hand (Furukawa et al., 2012). Electrophysiological manifestation of MVF with different hands is reflected in the intensity of ERS/ERD. While left hand MVF increases bilateral ERD in 8–10 Hz, left hand MVF results in reduced ERD in beta band 12–20 Hz on the ipsilateral (left) hemisphere (Bartur et al., 2015). This might affect not only ERS/ERD patterns but also a spatial distribution of domains. For example a large bilateral domain over BA6, D2_MVF_ related to motor planning might not be bilateral in case of left hand MVF.

Measure projection analysis creates domains using a similarity measure based on correlation. In the literature, fMRI rather than EEG was used to analyze functional connectivity between different areas of the cortex in BM and MVF tasks. In our study we found a strong presence of BA 6, covering both premotor and supplementary motor areas, responsible for motor planning in several functional domains. The fMRI studies from literature showed strong involvement of premotor and supplementary motor cortex (BA6) in functional networks present in both BM and MVF tasks (Gao et al., 2008; Hamzei et al., 2012), and strong involvement of supplementary motor areas during preparation for coordinated bimanual motor task (Welniarz et al., 2019). Hamzei et al. (2012) analyzed bimanual movements and showed increased functional connectivity based on dynamic causal modeling between the premotor cortices (both left and right) and the ipsilateral supplementary motor area. Based on the Granger causality analysis, Gao et al. (2008) found that the left and right supplementary motor cortices interact during bimanual movements, and that the supplementary motor area and cerebellum are active before the premotor cortex. In this study could not include the cerebellum and were not able to determine the direction of functional connectivity but we identified functional domains where BA6 of both left and right hemisphere were involved in both BM and MVF tasks. A study by Szameitat et al. (2012) compared a functional connectivity based on a psychophysiological interactions between the uni and bimanual motor imagination task. They found increased connectivity between the parietal and premotor areas within and between hemispheres during bimanual task. In our study we found for BM tasks a domain (D1_BM_) which encompasses both premotor and parietal areas on the left hemisphere.

Functionally distinctive cortical areas have characteristic ERS/ERD patterns, which for most domains are comparable between BM and MVF motor tasks. The largest differences between tasks exist in a domain functionally related to the awareness and planning of action of the left hand. This is of importance for rehabilitation of movement in stroke patients, who often have strong intracortical inhibition from the contralesional side, that impedes motor recovery (Jones and Adkins, 2015). MVF is typically recommended for patients who cannot move a paretic hand. However, the existence of additional domains related to motor planning of hand behind the mirror indicates that MVF might a useful sensory and motor priming strategy (Stoykov and Madhavan, 2015) in patients capable of bimanual therapy.

## Ethics Statement

Participants signed the informed consent form prior to taking part in the study. The study was approved by the University of Glasgow, College of Science and Engineering Ethical Committee. A member of research team provided a written consent to publish her photo.

## Author Contributions

SA-W conducted the experiments and analyzed data. FP contribute to the experimental design and paper writing. AS contributed to the data analysis and paper writing. AV designed the experiment, contributed to data analysis, and wrote the paper.

### Conflict of Interest Statement

The authors declare that the research was conducted in the absence of any commercial or financial relationships that could be construed as a potential conflict of interest.
